# Altered intestinal microbiota composition with epilepsy and concomitant diarrhea and potential indicator biomarkers in infants

**DOI:** 10.3389/fmicb.2022.1081591

**Published:** 2023-01-11

**Authors:** Tingting Liu, Fengan Jia, Ying Guo, Qi Wang, Xiaoge Zhang, Fan Chang, Yun Xie

**Affiliations:** ^1^Department of Pediatrics, Northwest Women’s and Children’s Hospital, Xi’an, China; ^2^Shaanxi Institute of Microbiology, Xi’an, China; ^3^Department of Clinical Laboratory, Second Affiliated Hospital of Xi’an Jiaotong University, Xi’an, China; ^4^Department of Clinical Laboratory, Northwest Women’s and Children’s Hospital, Xi’an, China

**Keywords:** epilepsy concomitant diarrhea, gut microbiota, assembly processes, association network, biomarkers

## Abstract

**Introduction:**

The diversity and dysregulation of intestinal microbiota is related to the pathology of epilepsy. Gut microbiota plays an important role in epilepsy, and regulating intestinal microbiota through exogenous intervention can alleviate symptoms. However, there are no studies about the effects of epilepsy-related diarrhea on gut microbiota.

**Methods:**

The diversity and dysregulation of intestinal microbiota is related to the pathology of epilepsy. Gut microbiota plays an important role in epilepsy, and regulating intestinal microbiota through exogenous intervention can alleviate symptoms. However, there are no studies about the effects of epilepsy-related diarrhea on gut microbiota. To evaluate changes in gut microbiota structure and composition in patients with epilepsy and associated diarrhea, the structure and composition of the fecal microbiota among patients with epilepsy (EP, 13 cases), epilepsy with diarrhea (ED, 13 cases), and probiotic treatments (PT, 13 cases), and healthy controls (CK, seven cases) were investigated and validated by utilizing high-throughput 16S rRNA sequencing.

**Results:**

The results showed that the α-diversity indexes indicated that richness and phylogenetic diversity had no significant differences among groups. However, the variation of β-diversity indicated that the structure and composition of intestinal microbiota were significantly different among the CK, EP, ED, and PT groups (permutational multivariate analysis of variance, *p*-value = 0.001). Normalized stochasticity ratio and β-nearest taxon index indicated that stochastic mechanisms exerted increasing influence on community differences with epilepsy and associated diarrhea. ED microbiome alterations include increased Proteobacteria and decreased Actinobacteria and Firmicutes at the phylum level. *Bifidobacterium* was the core microbe in CK, EP, and PT, whereas it decreased significantly in ED. In contrast, *Escherichia/Shigella* was the core microbe in CK and ED, whereas it increased significantly in ED (Tukey’s multiple comparisons test, adjusted *p*-value <0.05). The association network in CK has higher complexity and aggregation than in the other groups. The EP network indicated high connectivity density within each community and high sparsity among communities. The bacterial community network of the ED had a more compact local interconnection, which was in contrast to that of PT. The top 7 microbial amplicon sequence variant–based markers that were selected by machine learning to distinguish the groups of epilepsy, probiotic treatments, and healthy infants had stronger discrimination ability. In addition, ASVs_1 (*Escherichia/Shigella*) and ASVs_3 (*Bifidobacterium*) had the most importance in the recognition.

**Discussion:**

Our research finally showed that infants with epilepsy, epilepsy with diarrhea, and probiotic treatments exhibit substantial alterations of intestinal microbiota structure and composition, and specific intestinal strains are altered according to different clinical phenotypes and can therefore be used as potential biomarkers for disease diagnosis.

## 1. Introduction

Epilepsy is a chronic neurologic disorder characterized by an enduring predisposition to generate epileptic seizures, affecting >50 million people worldwide and 0.5–1% of children (Aaberg et al., 2017). Approximately 0.67% of children are diagnosed with epilepsy during the first 10 years of life, with the highest incidence rate observed during infancy (Eltze et al., 2013). Research has found that epilepsy is a symptom complex with multiple risk factors rather than a condition with a single expression and cause. A new cluster of clinical features resulted in the new classification of epileptic seizures and epilepsies (Thijs et al., 2019).

Accumulating evidence indicates that gut microbiota diversity and dysbiosis may be involved in the pathology of epilepsy. The gut microbiota plays a major role in epilepsy, and alteration or regulation through exogenous interventions may reduce or prevent epilepsy (Arulsamy et al., 2020). The gut–brain axis, which is the largest axis, has been recently shown to sense and react to dynamic ecosystem changes by converting microbiota-associated chemical cues from the environment into neuronal impulses, thus implicating a potential role of the gut microbiota in epileptogenesis (Yue et al., 2022). More evidence indicates that gut dysfunction/disorder is closely associated with the onset of and susceptibility to epilepsy, and some specific intestinal flora can function as gut biomarkers (Gong et al., 2020; Cui et al., 2022).

To date, various studies have primarily evaluated the role of alterations in the gut microbiome in epilepsy. These studies mainly focused on the changes in gut microbiota in patients with drug-resistant epilepsy (Gómez-Eguílaz et al., 2018; Peng et al., 2018) and the effect of a ketogenic diet on prognosis and gut microbiota (Lindefeldt et al., 2019; Ang et al., 2020). Few studies have focused on the syndrome and changes in gut microbiota in children, especially infants with epilepsy. Studies have shown that diarrhea can induce convulsions or cause them directly (Iflah et al., 2021). In the early clinical diagnosis, we found that some infants have unexplained diarrhea. And probiotic treatment reduced seizure frequency in epilepsy infants with diarrhea and significantly improved diarrhea. Here, for the first time, we conducted a detailed assessment of the gut microbiota in infants with epilepsy and diarrhea complications and examined the efficacy of probiotic adjuvant therapy in infants with epilepsy and diarrhea. Our study further revealed that the identified microbial signature can be used to evaluate the diagnosis of epilepsy syndromes.

## 2. Materials and methods

### 2.1. Study patient cohort and sample collection

The study was approved by the Ethics Committee of the Northwest Women’s and Children’s Hospital. A total of 20 infants (0–24 months) with epilepsy attending Northwest Women’s and Children’s Hospital were enrolled from May to June 2021 including healthy infants with negative control cases (CK group, seven cases), epilepsy group (EP group, 13 cases), epilepsy with diarrhea cases (ED group, 13 cases), and ED cases with additional probiotic (*Clostridium butyricum* MIYAIRI 588, CBM588) treatments (PT group, 13 cases). Oral administration of probiotic (CBM588, 1.0 × 10^6^ cfu/g, 0.5 g) was given twice a day for 2 weeks. All patients were from the same children’s ward and were breast-feeding. Patients were excluded if they have been administered antibiotics or probiotics within 3 months or had a known history of any other diseases. All patients with epilepsy were diagnosed for the first time without any drug therapies. Clinical information including age, gender, seizure types, and concomitant antiepileptic drugs (AEDs) used was registered separately. All infants’ fecal samples were collected as the first bowel movement upon admission for treatment using a validated stool collector. Samples were treated three times with liquid nitrogen and stored at −80°C as soon as possible until further analyses.

### 2.2. DNA extraction, amplification, and sequencing

DNA from different samples was extracted using the E.Z.N.A.® Stool DNA Kit (D4015, Omega, Inc., United States) according to the manufacturer’s instructions. The reagent, which was designed to uncover DNA from trace amounts of the sample, is effective for the preparation of the DNA of most bacteria. Nuclease-free water was used as the blank control. The total DNA was eluted in 50 μl of elution buffer and stored at −80°C until measurement in the PCR by LC-Bio Technology Co., Ltd., Hangzhou, Zhejiang Province, China.

The V3–V4 region of the prokaryotic (bacterial and archaeal) small-subunit (16S) rRNA gene was amplified with primers 341F (5′-CCTACGGGNGGCWGCAG-3′) and 805R (5′-GACTACHVGGGTATCTAATCC-3′; Takahashi et al., 2014). The 5′ ends of the primers were tagged with specific barcodes per sample and sequencing universal primers. PCR amplification was performed in a 25 μl total volume of reaction mixture containing 25 ng of template DNA, 12.5 μl of PCR Premix, 2.5 μl of each primer, and PCR-grade water to adjust the volume. The PCR conditions to amplify the prokaryotic 16S fragments consisted of an initial denaturation at 98°C for 30 s; 32 cycles of denaturation at 98°C for 10 s, annealing at 54°C for 30 s, and extension at 72°C for 45 s; and then final extension at 72°C for 10 min. The PCR products were confirmed with 2% agarose gel electrophoresis. Throughout the DNA extraction process, ultrapure water, instead of a sample solution, was used to exclude the possibility of false-positive PCR results as a negative control. The PCR products were purified by AMPure XT beads (Beckman Coulter Genomics, Danvers, MA, United States) and quantified by Qubit (Invitrogen, United States). The amplicon pools were prepared for sequencing using Agilent 2100 Bioanalyzer (Agilent, United States), and the size and quantity of the amplicon library were assessed using the Library Quantification Kit for Illumina (Kapa Biosciences, Woburn, MA, United States). The libraries were sequenced on the NovaSeq PE250 platform. The raw data from 46 samples were available from the Sequence Read Archive (SRA) under the accession number: PRJNA893837.

### 2.3. Sequence processing and taxonomic affiliation

Samples were sequenced on an Illumina NovaSeq platform according to the recommendations provided by LC-Bio. Paired-end reads were assigned to samples on the basis of their unique barcode and truncated by cutting off the barcode and primer sequence. The Usearch10 (Edgar, 2010) and Vsearch 2.8.1 (Rognes et al., 2016) data analysis pipelines were used for 16S rRNA data analysis. Forward and reverse reads were joined, assigned to samples on the basis of barcodes, and truncated by the removal of the barcode and primer sequences. Quality filtering was performed on joined sequences. Sequences that did not fulfill the following criteria were discarded: no ambiguous bases and expected errors per base rate >0.01 and dereplicated and singleton sequences (size <8). Then, sequences were clustered into amplicon sequence variants (ASVs) using the Unoise3 sequence variant algorithm (Edgar, 2018; Knight et al., 2018), and chimeric sequences were simultaneously removed. Sequences were grouped using the clustering program Vsearch 2.8.1 against the Ribosomal Database Project^1^ and preclustered at 97% sequence identity. The classifier (Wang et al., 2007) was used to assign taxonomic categories to all ASVs at a confidence threshold of 0.8.

### 2.4. Data analysis

All statistical analyses were performed using R software (v4.1.2).^2^ The α-diversity was evaluated using the Chao1 and Faith’s phylogenetic diversity (Faith’s PD) indexes on the basis of the relative abundance *via* the “vegan” package (Oksanen et al., 2019) in R. Phylogenetic tree analysis of soil was constructed using cluster_agg of Usearch10 (Edgar, 2010) with the Bray–Curtis distances. The α-diversity indexes were calculated with a one-way analysis of variance and Tukey’s multiple comparisons test. Principal coordinate analysis (PCoA) plots were generated from a Bray–Curtis dissimilarity matrix of ASVs, and permutational multivariate analysis of variance analysis was performed using the “vegan” package (Oksanen et al., 2019).

The “NST” package was used to calculate the stochastic ratio (normalized stochasticity ratio, NST) to estimate the relative importance of stochasticity in shaping community structure (Ning et al., 2019). Furthermore, to explore the structure of bacterial community assembly processes by deterministic or stochastic processes, the β-nearest taxon index (βNTI; Stegen et al., 2012) was calculated using the “picante” package (version 1.8.2). The βNTI was calculated for pairwise phylogenetic turnover among communities to estimate the proportion of determinism. |βNTI| > 2 indicates that observed turnover between a pair of communities is governed primarily by determinism, which could be divided into homogeneous selection (βNTI < −2) and heterogeneous selection (βNTI > +2). On the contrary, |βNTI| < 2 indicates that observed differences in phylogenetic composition between a pair of communities are governed primarily by stochasticity. Then, the relative contributions of stochastic processes were estimated using the Bray–Curtis-based Raup–Crick matrix (RCbray). If |RCbray| > 0.95, community assembly is considered significantly dominated by dispersal either by homogenizing dispersal (RCbray < −0.95) or by dispersal limitation (RCbray > +0.95). However, if |RCbray| < 0.95, then the community is an undominated process.

For taxonomic analysis, the “ggplot2” package was used to draw the alluvial diagram at the phylum and genus levels to check the relative species abundance. The core bacterial taxa of different groups were calculated using the “miaverse” package (Amezquita et al., 2020), and the samples with a relative abundance threshold value greater than 0.01% were identified to use the core function.

The association network analysis was used to construct and display Spearman’s correlation coefficient of indicator species. First, the “indicspecies” package (Cáceres and Legendre, 2009) was used to obtain indicator species. Indicators with *p*-value <0.05 and relative abundance >0.05% were selected as the initial ASVs for the construction of correlation networks. The correlation network analysis was performed using 0.8 as the correlation coefficient cutoff. The final network included significant edges (*p* < 0.05) in Spearman’s correlation used. The major topological properties were also calculated using Gephi (v0.92).^3^

The random forest models were constructed using the “randomForest” package (Breiman, 2001). The nested cross-validation was performed to filter the important predictive ASVs. Furthermore, the mean decrease accuracy, mean decrease Gini, and relative abundance of important predictive ASVs were calculated to show the accuracy and importance in different groups.

## 3. Results

### 3.1. Study population characteristics

A total of 13 patients with epilepsy (EP), 13 patients with epilepsy with diarrhea (ED), 13 ED patients with additional probiotic treatments (PT), and seven healthy infants (CK) were enrolled. To reduce the impact of diet on the gut microbiota, all patients were from the same children’s ward and were breast-feeding. The average age and sex ratios were similar among the three groups. Oxcarbazepine was the most frequently used drug, followed by topiramate and valproate (Table 1).

**Table 1:** Characteristics of the patients included in the study.

	CK (*n* = 7)	EP (*n* = 13)	ED (*n* = 13)	PT (*n* = 13)
Age (m, means ± SD)	4.9 ± 2.5	4.6 ± 2.7	4.2 ± 2.4	4.5 ± 2.6
Gender				
Male	3 (42.86)	7 (53.85)	6 (46.15)	7 (53.85)
Female	4 (57.14)	6 (46.15)	7 (53.85)	6 (46.15)
Seizure types (*n*, percentage)				
Generalized	–	8	8	7
Partial	–	3	4	4
West syndrome	–	2	1	2
Concomitant AEDs (*n*, percentage)				
Oxcarbazepine, OXC	–	6 (46.15%)	7 (53.85%)	7 (53.85%)
Topiramate, TPM	–	4 (30.77%)	5 (38.46%)	4 (30.77%)
Valproate, VPA	–	2 (15.39%)	2 (15.39%)	3 (23.08%)
Lamotrigine, LTC	–	2 (15.39%)	2 (15.39%)	2 (15.39%)
Adrenocorticotropic hormone, ACTH	–	2 (15.39%)	1 (7.69%)	2 (15.39%)

m represents months, which was rounded down. SD represents standard deviation. *n* represents the sample numbers. Data are reported as means ± SD for continuous variables and percentage for nonparametric data. CK, healthy controls; EP, epilepsy; ED, epilepsy with diarrhea; PT, probiotic treatments.

### 3.2. Bacterial gut microbial diversity of healthy individuals differed from those of diseased infants

After filtering and chimera removal, 46 samples yielded a total of 3,545,269 high-quality sequences, with an average of 76,735 sequences for each sample. High-quality sequences were clustered into 1,928 microbial ASVs by using Unoise3 sequence identity.

**Figure 1:**
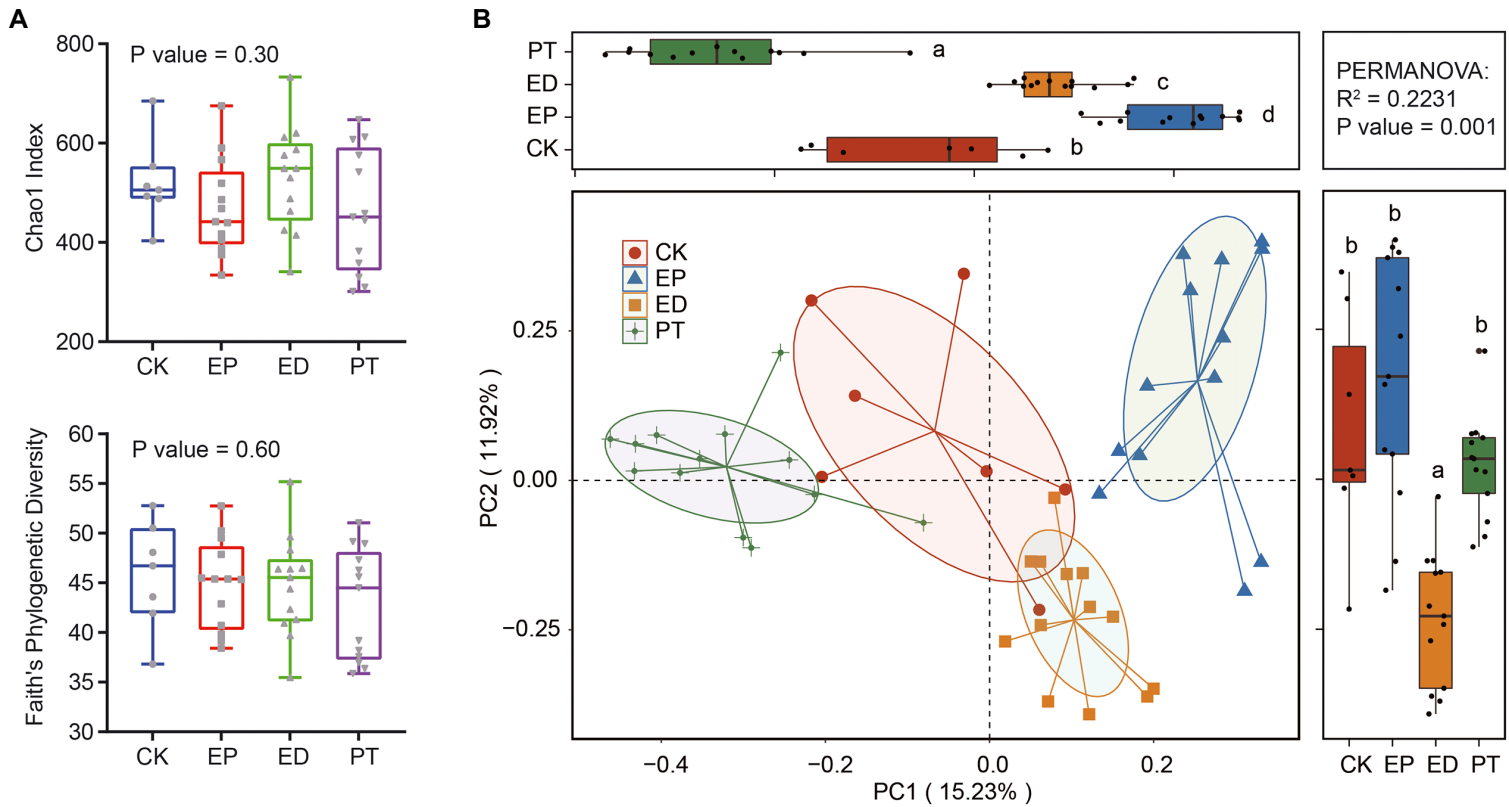
Comparison of gut microbiome structure at the amplicon sequence variant (ASV) level in epilepsy (EP), epilepsy with diarrhea (ED), additional probiotic treatments (PT), and healthy infants (CK). **(A)** Chao1 and Faith’ PD indexes in different groups. **(B)** The principal coordinate analysis (PCoA) plot based on the Bray–Curtis distance metric of ASVs in different groups.

The α-and β-diversity in the fecal samples of infants with epilepsy and diarrhea and healthy infants were estimated. The α-diversity analyses indicated that the richness (measured by the Chao1 index) and phylogenetic diversity (measured by Faith’s PD index) had no significant differences among groups (Figure 1A). There was no significant difference in α-diversity between different types of Seizure and different AED treatment (Supplementary Figure S1).

Furthermore, the variation of β-diversity was assessed on the basis of the Bray–Curtis dissimilarity matrix of ASVs. PCoA indicated that patients with epilepsy (EP) and epilepsy with diarrhea (ED) and ED patients with additional probiotic treatments (PT) harbored distinct species compositions as compared with the healthy controls (Figure 1B). Collectively, PCoA contributed 27.15% variation to the bacterial community composition (PC1, 15.23%; PC2, 11.92%). The gut bacterial community structures among all groups were clustered along the PC1 axis. In particular, the ED group was clustered along the PC2 axis. The permutational multivariate analysis of variance supported the finding that the gut community structure in different groups indicated a significant association with the variation in species composition (*p*-value = 0.001).

### 3.3. Assembly processes of bacterial gut microbial communities

**Figure 2:**
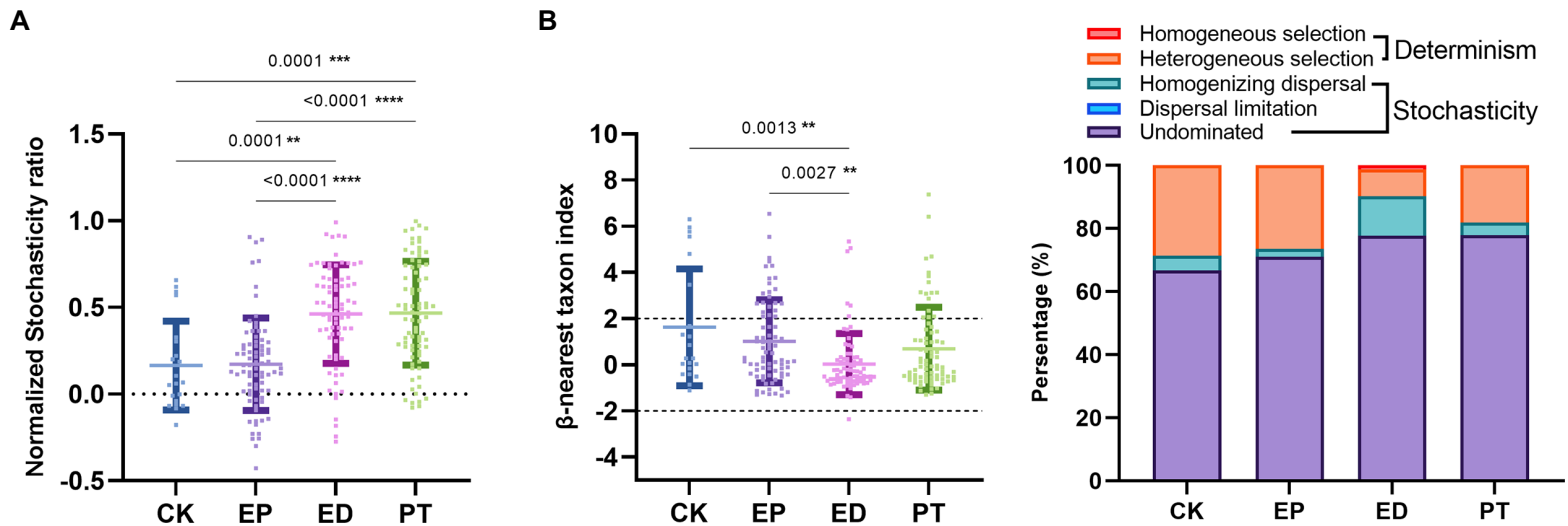
Comparisons to determine assembly mechanisms of intestinal microbiota among individuals within different groups (EP, ED, PT, and CK). **(A)** Normalized stochasticity ratio (NST) in different groups. **(B)** β-Nearest taxon index (βNTI) in different groups and dynamics of the relative importance of different community assembly processes. Asterisks indicated comparisons with an adjusted *p*-value <0.05, based on Tukey’s multiple comparisons test. CK, healthy controls, EP, epilepsy; ED, epilepsy with diarrhea; PT, probiotic treatments.

To quantify the relative importance of deterministic processes in shaping gut microbial community succession, NST was calculated. Overall, determinism was the predominant mechanism. The importance of stochasticity contributed to community variations at 0.17 ± 0.26, 0.17 ± 0.27, 0.46 ± 0.28, and 0.46 ± 0.30 for the CK, EP, ED, and PT groups, respectively (Figure 2A and Supplementary Table S2). Another null model approach that constituted taxonomic and phylogenetic matrices was used to further disentangle different assembly processes and quantify their relative importance. Across the four subjects’ groups, the βNTI of phylogenetic dissimilarity between a pair of communities was 1.63 ± 2.53, 1.02 ± 1.80, 0.03 ± 1.33, and 0.70 ± 1.80, and there was a concomitant increase in the stochasticity percentage of processes (Figure 2B and Supplementary Table S2). In particular, it affected the reduction of heterogeneous selection and the increase of homogenizing dispersal (Figure 2B), demonstrating that the relative contribution of stochasticity played a more important role than determinism. Overall, this indicated that stochastic mechanisms exerted increasing influence on community differences with epilepsy and related symptoms. Meanwhile, heterogeneous selection was increased in the PT group after the additional probiotic treatment.

### 3.4. Taxonomic compositions and core microbiota of epilepsy, epilepsy with diarrhea, and healthy infants

**Figure 3:**
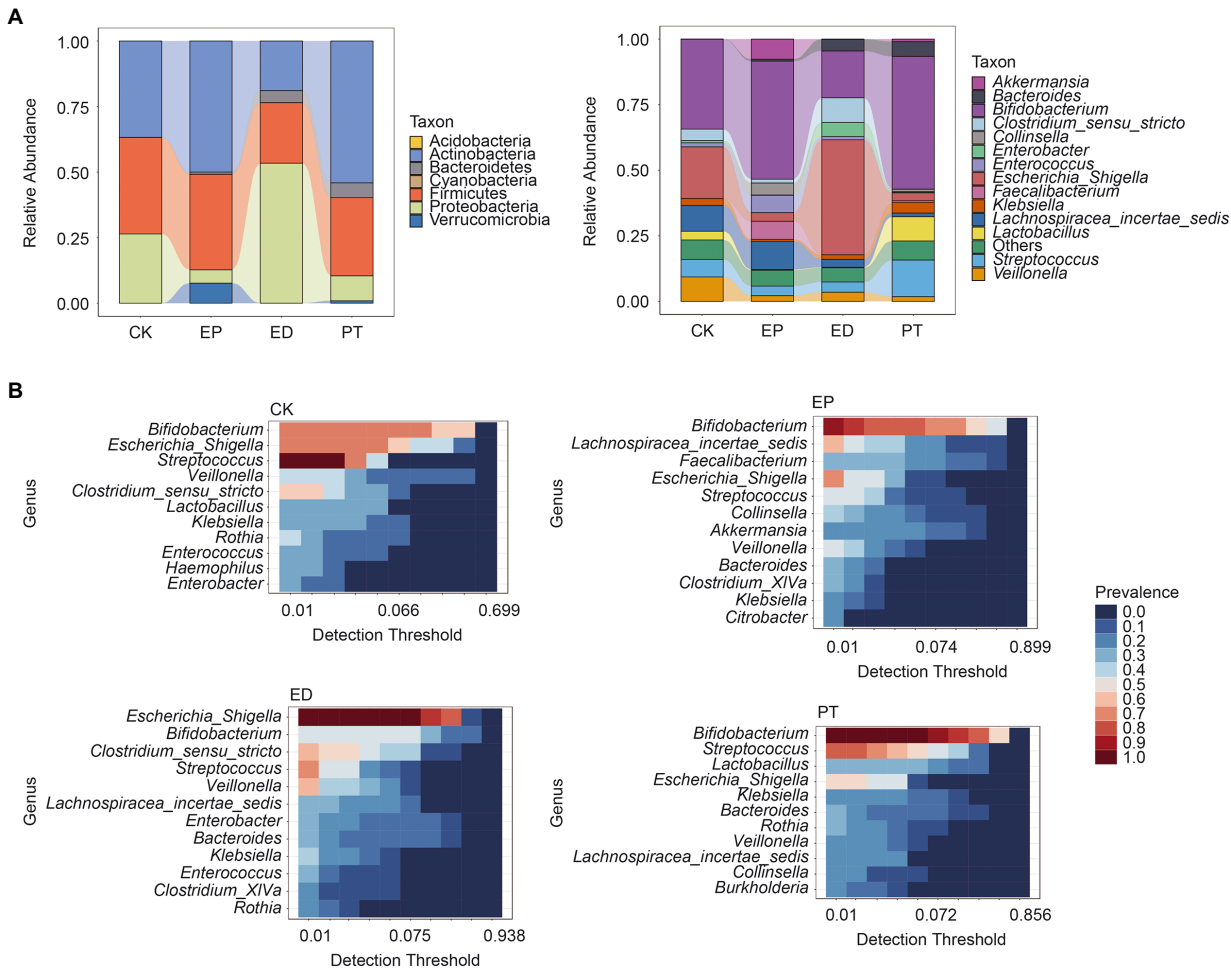
Relative abundance and core microbiota among individuals within different groups. **(A)** The relative abundance at the phylum and genus levels in different groups. **(B)** The core microbiota at the genus level in different groups. *Detection threshold* represents the relative abundance.

The relative abundance of bacterial community composition at the phylum and genus levels is shown in Figure 3A. Acidobacteria, Firmicutes, and Proteobacteria were revealed as the dominant phyla in all groups. The relative abundance of Proteobacteria was significantly higher, and that of Actinobacteria and Firmicutes was significantly lower (Tukey’s multiple comparisons test, adjusted *p*-value <0.05) in the ED group than in the other groups. Meanwhile, the relative abundance of Proteobacteria in the EP and PT groups was significantly lower (adjusted p-value <0.05) than that in the CK and ED groups (Figure 3A and Supplementary Table S3).

At the genus level, the relative abundance of *Bifidobacterium* was higher (adjusted p-value <0.05) in the EP and PT groups than in the CK and ED groups but was the lowest in the ED group. Meanwhile, *Bifidobacterium* was the core microbe in the CK (prevalence = 0.71), EP (prevalence = 0.92), and PT (prevalence = 1.00) groups. *Escherichia/Shigella* has the highest relative abundance in the ED group and was the core microbe in the CK (prevalence = 0.71) and ED (prevalence = 1.00) groups (Figure 3B and Supplementary Table S3). Also, *Streptococcus* was the core microbe in the CK, ED, and PT groups, with a prevalence of 1, 0.70, and 0.77, respectively.

### 3.5. Microbiota association network of epilepsy, epilepsy with diarrhea, and healthy infants

**Figure 4:**
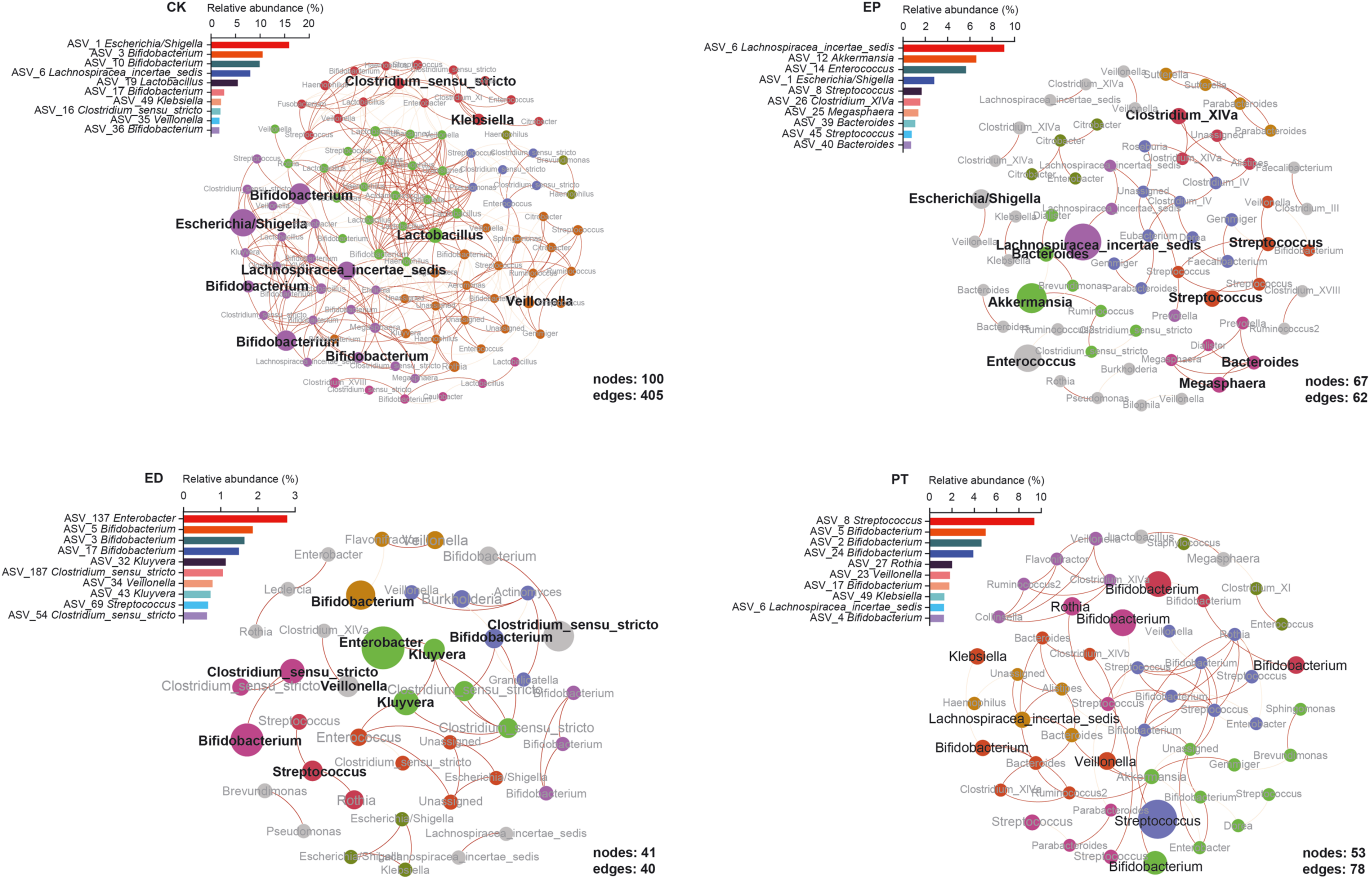
Epilepsy (EP) and concomitant diarrhea (ED) attenuated the network interactions of the gut bacterial taxa, and special microbiota occupied the keystone interaction nodes in microbiome networks.

To examine the community structure of the microbiota, the microbiota correlation networks of different groups were created. The ASVs with relative abundance >0.05% correlation are shown in Figure 4. Using Spearman’s rank to calculate the correlation coefficient, only the absolute value higher than 0.8 could be shown. Red edges indicate the co-occurrence, whereas yellow edges indicate mutual exclusion. The red-to-yellow color transition represents the degree of correlation. The size of the nodes represents the average relative abundance of ASVs, and the same color represents the same cluster within the different groups. The relative abundance of the top 10 ASVs and positions in the networks are also shown. Meanwhile, the local properties of the networks that were analyzed included average degree distribution, average path length, average clustering coefficient, and modularity (Supplementary Table S4).

From a global perspective, the network in the different groups was divided into the main components. The network of the CK group has more nodes (100) and edges (405) and higher complexity than the other three groups. The modules in each group were highlighted with community modularity algorithm, and 6, 18, 12, and 8 modules were clustered in different groups. The average degree distribution of the CK group was the highest (8.100), which represented stronger network stability (Figure 4, CK, Supplementary Table S4). The ED group had the highest average clustering coefficient (0.609), and the relative frequency could define the clustering of the network, which reflected the characteristics of aggregation of the network. The network of the ED group had the smallest average path length (2.339), indicating more compact local interconnection and denser bacterial communities (Figure 4, ED, Supplementary Table S4). The high modularity (0.858) of the EP network indicated high connectivity density within each community while high sparsity among communities (Figure 4, EP, Supplementary Table S4). The PT group network had the largest average path length through probiotic treatments. Compared with the ED group, the PT group’s number of nodes and edges was increased, while the average clustering coefficient, modularity, and modules were reduced (Figure 4, PT, Supplementary Table S4).

First, no genus was found in all groups*. Bifidobacterium* had the highest relative abundance in the networks of the CK (24.78%), ED (5.00%), and PT (16.61%) groups but was not observed in the EP network. Instead, *Lachnospiraceae* incertae sedis had the highest relative abundance in EP (9.08%) but was not observed in the ED network. *Streptococcus* was found in the EP (2.46%), ED (2.02%), and PT (9.38%) groups but not in the CK group. Also, *Veillonella* was found only in the CK (1.73%), ED (0.79%), and PT (1.82%) groups (Figure 4 and Supplementary Table S5). Second, each group had its unique bacterial species. The EP network had the most unique species, including *Akkermansia*, *Bacteroides*, *Clostridium* XlVa, *Enterococcus*, and *Megasphaera*. *Enterobacter* and *Kluyvera* were only observed in the ED network. *Lactobacillus* was only found in CK, and *Rothia* was only observed in the PT group. All common or endemic species with high relative abundance were at the important nodes in their respective clusters (Figure 4).

### 3.6. Identification and validation of microbial ASV-based markers for epilepsy and concomitant diarrhea

**Figure 5:**
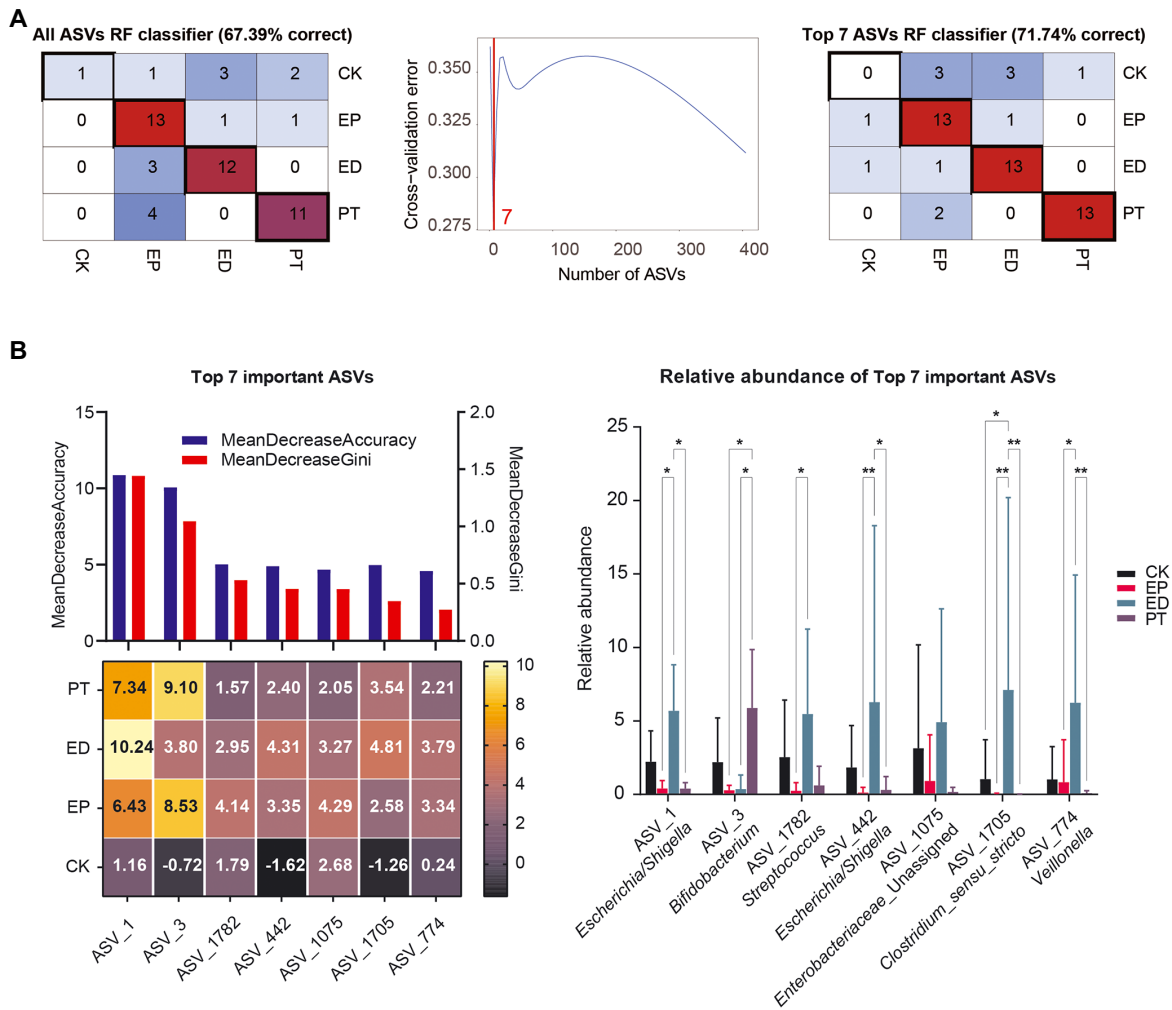
Identification of microbial markers of epilepsy and concomitant diarrhea by random forest models. **(A)** A 10-fold cross-validation on a random forest model was performed to detect unique amplicon sequence variant (ASV)–based markers. **(B)** The mean decrease accuracy, mean decrease Gini, importance, and relative abundance of the top 7 ASVs.

To evaluate the classification power of fecal bacteria markers for epilepsy and concomitant diarrhea, a random forest classifier (RFC) model was constructed. A 10-fold cross-validation on a random forest model was performed to detect unique ASV-based markers. The analysis identified the top 7 differentially abundant markers as the optimal marker set. The accuracy of the model was then calculated using the identified seven ASV-based markers. It was found that using the top 7 optimal markers as the set to identify different groups improved the RFC model’s accuracy (67.39–71.74%; Figure 5A).

Also, the mean decrease accuracy, mean decrease Gini, and importance of ASVs are shown in Figure 5B. ASVs_1 (*Escherichia/Shigella*) and ASVs_3 (*Bifidobacterium*) had the most importance in the recognition of epilepsy and concomitant diarrheal diseases. Meanwhile, ASV_1782 (*Streptococcus*), ASV_442 (*Escherichia/Shigella*), ASV_1075 (*Enterobacteriaceae_*Unassigned), ASV_1705 (*Clostridium sensu stricto*), and ASV_774 (*Veillonella*) also played a key role in the recognition of different disease groups from the importance scores in different groups. Interestingly, some markers in the CK group had negative importance scores, indicating a more random distribution of gut bacterial communities in healthy infants.

From the changes in the relative abundance of ASVs, it could be found that ASV1, ASV_1782, ASV_442, ASV_1075, ASV_1705, and ASV_774 had the same pattern: the average relative abundance was the highest in the ED group, followed by the CK group, and the relative abundance was lower in the EP and PT groups. In contrast, ASV3 had the highest mean relative abundance in the PT group, suggesting that probiotic treatment may increase the relative abundance of *Bifidobacterium*.

## 4. Discussion

### 4.1. Effects of epilepsy and concomitant diarrhea on the gut microbiota of infants

In infant epilepsy research, early evaluation and unclear mechanisms of complications remain a challenge (Elliott et al., 2019; Specchio and Curatolo, 2021). In recent years, microbiota signatures have received extensive attention because of their excellent performance in early detection and prognostic assessment in various disease areas (Dahlin and Prast-Nielsen, 2019; Lindefeldt et al., 2019). Meanwhile, a growing number of studies have found a significant correlation among microbiome characteristics and epilepsy-related diseases (Alderton, 2018; Gómez-Eguílaz et al., 2018). Our study revealed the structure and changes of the gut microbiota in infants with epilepsy and unexplained diarrhea. A synergistic process of gut microbiota assembly in children with epilepsy was described. Furthermore, we elucidated the phylum-and genus-level effects of diarrhea on the gut microbiota of children with epilepsy and reported the validation of AVS-based epilepsy microbial markers.

Our findings indicated that epilepsy and diarrhea were associated with the β-but not α-diversity of infants’ gut microbiota. Although Chao1 and Faith’s PD indexes fluctuated, the large range of α-diversity indicated that despite consistent feeding patterns, there were large inter-individual differences in intestinal microbiota, which may come from the microbiota of breast milk (Ho et al., 2018). In contrast, the β-diversity indexes (Bray–Curtis dissimilarity) of gut microbiota were significantly different among different groups. This indicated that there were significant differences in the number and abundance of the infant gut microbiota among the groups. Such results have been reported in adult and animal gut microbiota studies (Li et al., 2016; de la Cuesta-Zuluaga et al., 2019).

### 4.2. Assembly process of epilepsy and concomitant diarrhea based on the gut microbiota of infants

Previous studies have found that the gut microbiota assembly process may affect the gastrointestinal microbiota during early childhood (Sprockett et al., 2018). In our study, the composition of the intestinal microbiota was used to assess the strength of the assembly process. Our findings indicated that the stochasticity of intestinal microbiota (characterized by NST) in infants with epilepsy and diarrhea (ED group) significantly increased and that probiotic treatment (PT group) could not alleviate the stochasticity distribution. Meanwhile, our study addressed the ecological processes underlying the assembly of microbes in epilepsy and diarrhea of infants. Stochasticity had always dominated the assembly process of intestinal microbiota in infants, and this trend was more significant in children with epilepsy and diarrhea (characterized by βNTI). Undominated processes, including diversification and drift, have the highest proportion among stochasticity in all groups. This finding was consistent with that of Seki, who observed that the gut microbiota of extremely premature infants was directed by ecological drift (Seki et al., 2022). Homogenizing dispersal was the most influential over the presence/absence of taxa (Martínez et al., 2015). Our study also showed that the homogenizing dispersal process of gut microbiota was significantly increased in infants with epilepsy and diarrhea (ED group), indicating that the intestinal species of the ED group, compared with other groups, had changed significantly.

### 4.3. Effects of epilepsy and concomitant diarrhea on intestinal microbiota species and association network in infants

Our results indicated that some phyla, including Actinobacteria and Firmicutes, were less abundant in the ED group and that Proteobacteria was more abundant in the ED group. Among them, *Bifidobacterium* belonging to the Actinobacteria phylum decreased in relative abundance, whereas *Escherichia/Shigella* belonging to the Proteobacteria phylum increased significantly. The current studies have indicated a decrease in Actinobacteria and an increase in Firmicutes in patients with epilepsy (Arulsamy et al., 2020). However, in our study, the relative abundance of Actinobacteria and Firmicutes did not change significantly in infants with epilepsy alone but decreased significantly in the group of infants with epilepsy with diarrhea. These infants exhibited typical characteristics of early developmental stages such as unstable community structure and low microbiome maturation (Xiao et al., 2021). This may be the main reason for the special intestinal microbiota structure of infants.

The increase in *Bifidobacterium* in infants with epilepsy observed herein was in agreement with findings from studies of patients with drug-resistant epilepsy and refractory epilepsy (Ang et al., 2020; Gong et al., 2020). Evidence had indicated that *Bifidobacterium* was associated with the prevention of drug-resistant epilepsy and activation of anti-allergic mechanisms in children (Cukrowska et al., 2020; Dahlin et al., 2022). As functional bacteria that digest complex carbohydrates, *Bifidobacterium* might play a potential role in the pathogenesis of epilepsy (Wang et al., 2016). Changes in *Bifidobacterium* lead to disturbances in carbohydrate metabolism and might increase the risk of epilepsy (Lindefeldt et al., 2019). In our study, the relative frequencies of *Bifidobacterium* were significantly reduced in infants with epilepsy and diarrhea. Meanwhile, *Bifidobacterium* was not observed in the main clusters of the association network in infants with epilepsy. The potential role of *Bifidobacterium* in the gut microbiota with epilepsy and diarrhea-induced gut microbiota reduction requires further investigation.

*Escherichia/Shigella* is a potential enteric pathogen involved in the pathogenesis of intestinal diseases (Wang et al., 2012; Radhakrishnan et al., 2022), which releases lipopolysaccharides and might induce a variety of inflammatory responses lipopolysaccharides are released in the gut. Meanwhile, *Escherichia/Shigella* toxins might cause encephalopathy (Lee et al., 2020b). In our study, *Escherichia/Shigella* was increased significantly in infants with epilepsy and diarrhea (ED) but decreased significantly in infants with epilepsy alone (EP) as compared with healthy infants. Our study also showed that *Escherichia/Shigella* had the highest relative abundance in ED, but it did not play an important position in the association network. These results relatively differed from those of another study (Cui et al., 2022). Our findings also suggested that intestinal microbiota, such as *Bifidobacterium* and *Escherichia/Shigella*, could be used as potential microbial markers to classify possible subtypes of epileptic diseases.

Previous studies have found that *Streptococcus* infection increased the risk of chronic diseases such as cerebral palsy and epilepsy (Lin et al., 2019; Yeo et al., 2019). Our findings indicated that treatment with CBM588 did significantly reduced *Escherichia/Shigella* in infants with epilepsy and diarrhea (PT), but the relative abundance of *Streptococcus* increased. In the PT association network, *Streptococcus* had the highest relative abundance and was an important cluster that occupied the center of the network.

The relative abundance of *Clostridium* in the PT group did not significantly increase in spite of CBM588 intervention. Intestinal microenvironment changes caused by diarrhea may make clostridium difficult to colonize (Takahashi et al., 2016). We hypothesized that CBM588 produces butyrate and that its fermented product D-lactate provides energy for colonic epithelial cells and plays an important role in epithelial barrier integrity and immune modulation, altering the intestinal microenvironment and making it suitable for *Bifidobacterium* like ASVs_3 proliferation (Wang et al., 2014). Human gut microbiota is a dynamic and complex microbial system linked to pathogen colonization resistance and immune system regulation (Bäumler and Sperandio, 2016). Our study suggested that single probiotics might have some drawbacks as a potential intestinal regulator. The microbiota might be viewed as potential therapeutics for regulating the intestinal microenvironment to treat various diseases (Vrancken et al., 2019).

### 4.4. Classification and potential diagnostic effects of intestinal biomarkers in infants with epilepsy and concomitant diarrhea

Machine learning has been demonstrated to have advantages in classifying and predicting epileptic diseases (Gong et al., 2020; Dahlin et al., 2022). Our study showed that even with complications and probiotic treatment, the random forest algorithm could still identify ASV-based marks and accurately distinguish different study cohorts. Epileptic disease–associated microbial dysbiosis was characterized by changes in the abundance of some ASVs. The groups with epilepsy, epilepsy with diarrhea, and probiotic treatment were differentiated using the top 7 taxa on the basis of RFC. Moreover, our results indicated that these microbial markers improved the accuracy of classification. As a result, the gut microbiota could be used to accurately identify patients. Although the study represented a new method for the diagnosis of epilepsy, large samples are still needed to verify its accuracy.

The pathogenesis of epilepsy is complex, and more than 30% of patients suffer from refractory epilepsy that cannot be controlled with drug therapy (Beghi et al., 2015; Kobow and Blümcke, 2018). These conditions pose a challenge to the diagnosis and treatment of epilepsy. As an efficient and non-invasive diagnostic method, the detection of gut microbiota is crucial to infants with epilepsy. At the same time, gut-microbiota-based biomarkers are important for the differential diagnosis, prognosis and treatment monitoring of epilepsy (Gong et al., 2020; Lee et al., 2020a). However, our study has some limitations. First, because this was an association study, no causal relationship could be drawn from it. Epilepsy and diarrhea complications could not be directly linked to the gut microbiome. Second, 16S rRNA sequencing was not sufficient to reveal all possible factors and relationships that might have influenced disease status at the species or strain level (Şafak et al., 2020). The application of shotgun metagenomic, transcriptomic, and metabolomic technologies may reveal minor network interference in the genes of the gut microbiome regarding their expression and the presence of metabolites (Dahlin and Prast-Nielsen, 2019). Therefore, future research should consider the functional effects of changes in the gut microbiota on disease initiation and complications, which will reveal the effects of the “gut–brain axis” mechanism on infant epilepsy.

## 5. Conclusion

The gut microbial diversity and structure of intestinal microbiota was significantly different in infants with different epilepsy symptoms. The structure and composition of intestinal microbiota were significantly different among the healthy and epilepsy infants’ groups. That stochastic mechanisms applied increasing influence to community differences with epilepsy and related diarrhea infants. In epilepsy with concomitant diarrhea, microbiome alterations include increased Proteobacteria and decreased Actinobacteria and Firmicutes. *Bifidobacterium* and *Escherichia/Shigella* were the core microbe in healthy and epilepsy with concomitant diarrhea infants. The association network in healthy infants has higher complexity and aggregation than the others. The epilepsy infants network indicated the high connectivity density within each community while the high sparsity between communities. While the network of the epilepsy with concomitant diarrhea infants had more compact local interconnection. Top 7 microbial ASVs-based markers that were selected by machine learning to distinguish the groups of epilepsy, probiotics treatments and healthy infants had stronger discrimination ability. And ASVs_1 (*Escherichia/Shigella*) and ASVs_3 (*Bifidobacterium*) had the most importance in the recognition.

Our research finally showed that infants with epilepsy, epilepsy with diarrhea and probiotics treatments exhibit substantial alterations of intestinal microbiota structure and composition, and specific intestinal strains are altered according to different clinical phenotypes and can therefore be used as potential biomarkers for disease diagnosis.

## Data availability statement

The datasets presented in this study can be found in online repositories. The names of the repository/repositories and accession number(s) can be found in the article/Supplementary material.

## Ethics statement

The studies involving human participants were reviewed and approved by the Ethics Committee of Northwest women’s and children’s hospital. Written informed consent to participate in this study was provided by the participants’ legal guardian/next of kin.

## Author contributions

YX, FC, TL, and FJ conceived and designed the experiments. TL and YG contributed to sampling. TL, YG, QW, and XZ carried out the experimental work and clinical data collection. FC, FJ, YX, and QW performed the data analysis and interpretation. TL, FJ, FC, and YX wrote and/or reviewed the manuscript. All authors contributed to the article and approved the submitted version.

## Funding

This study was supported by Shaanxi Provincial Health Scientific Research Fund Project (2018E014).

## Conflict of interest

The authors declare that the research was conducted in the absence of any commercial or financial relationships that could be construed as a potential conflict of interest.

## Publisher’s note

All claims expressed in this article are solely those of the authors and do not necessarily represent those of their affiliated organizations, or those of the publisher, the editors and the reviewers. Any product that may be evaluated in this article, or claim that may be made by its manufacturer, is not guaranteed or endorsed by the publisher.

## Footnotes

^1^ http://rdp.cme.msu.edu/

^2^ http://www.r-project.org/

^3^ https://gephi.org
